# Golgi Fragmentation in ALS Motor Neurons. New Mechanisms Targeting Microtubules, Tethers, and Transport Vesicles

**DOI:** 10.3389/fnins.2015.00448

**Published:** 2015-12-08

**Authors:** Georg Haase, Catherine Rabouille

**Affiliations:** ^1^Centre National de la Recherche Scientifique and Aix-Marseille Université UMR 7289, Institut de Neurosciences de la TimoneMarseille, France; ^2^The Department of Cell Biology, Hubrecht Institute of the Royal Netherlands Academy of Arts and Sciences and University Medical Center UtrechtUtrecht, Netherlands

**Keywords:** Golgi fragmentation, neurodegeneration, ALS, microtubules, SOD1, TDP-43, TBCE, C9orf72

## Abstract

Pathological alterations of the Golgi apparatus, such as its fragmentation represent an early pre-clinical feature of many neurodegenerative diseases and have been widely studied in the motor neuron disease amyotrophic lateral sclerosis (ALS). Yet, the underlying molecular mechanisms have remained cryptic. In principle, Golgi fragmentation may result from defects in three major classes of proteins: structural Golgi proteins, cytoskeletal proteins and molecular motors, as well as proteins mediating transport to and through the Golgi. Here, we present the different mechanisms that may underlie Golgi fragmentation in animal and cellular models of ALS linked to mutations in SOD1, TARDBP (TDP-43), VAPB, and C9Orf72 and we propose a novel one based on findings in progressive motor neuronopathy *(pmn)* mice. These mice are mutated in the TBCE gene encoding the cis-Golgi localized tubulin-binding cofactor E, one of five chaperones that assist in tubulin folding and microtubule polymerization. Loss of TBCE leads to alterations in Golgi microtubules, which in turn impedes on the maintenance of the Golgi architecture. This is due to down-regulation of COPI coat components, dispersion of Golgi tethers and strong accumulation of ER-Golgi SNAREs. These effects are partially rescued by the GTPase ARF1 through recruitment of TBCE to the Golgi. We hypothesize that defects in COPI vesicles, microtubules and their interaction may also underlie Golgi fragmentation in human ALS linked to other mutations, spinal muscular atrophy (SMA), and related motor neuron diseases. We also discuss the functional relevance of pathological Golgi alterations, in particular their potential causative, contributory, or compensatory role in the degeneration of motor neuron cell bodies, axons and synapses.

## Introduction

Amyotrophic Lateral Sclerosis (ALS) is a severe neurodegenerative disease characterized by progressive degeneration of motor neurons in spinal cord, brainstem and cerebral cortex and of their corresponding axons in the corticospinal tract and in peripheral nerves. Degeneration of motor axons and loss of neuromuscular synapses leads to denervation of skeletal muscle fibers which causes progressive muscle weakness and paralysis and becomes fatal when it reaches critical muscle groups, usually within 2–5 years after disease onset (Robberecht and Philips, 2013).

ALS can be caused by mutations in more than 20 genes, including the major ones SOD1, TARDBP (TDP-43), FUS and C9ORF72, or manifest as apparently sporadic form. In ALS motor neurons, many cellular functions are altered as illustrated by defects in nucleocytoplasmic transport (Freibaum et al., 2015; Jovicic et al., 2015; Zhang et al., 2015), in processing of mRNAs (Lourenco et al., 2015) and miRNAs (Emde et al., 2015), in formation of stress granules (Li et al., 2013), ER stress (Matus et al., 2013), mitochondrial dysfunction (Pasinelli et al., 2004), and alterations in almost all steps of membrane traffic. For instance, autophagy (Ferrucci et al., 2011; Song et al., 2012; Majcher et al., 2015), endocytosis (Rusten and Simonsen, 2008), and secretory function (Gonatas et al., 1998; Nassif et al., 2010) have been shown to be affected. In this review, we will focus on structural and functional alterations of the Golgi apparatus.

**Figure 1:**
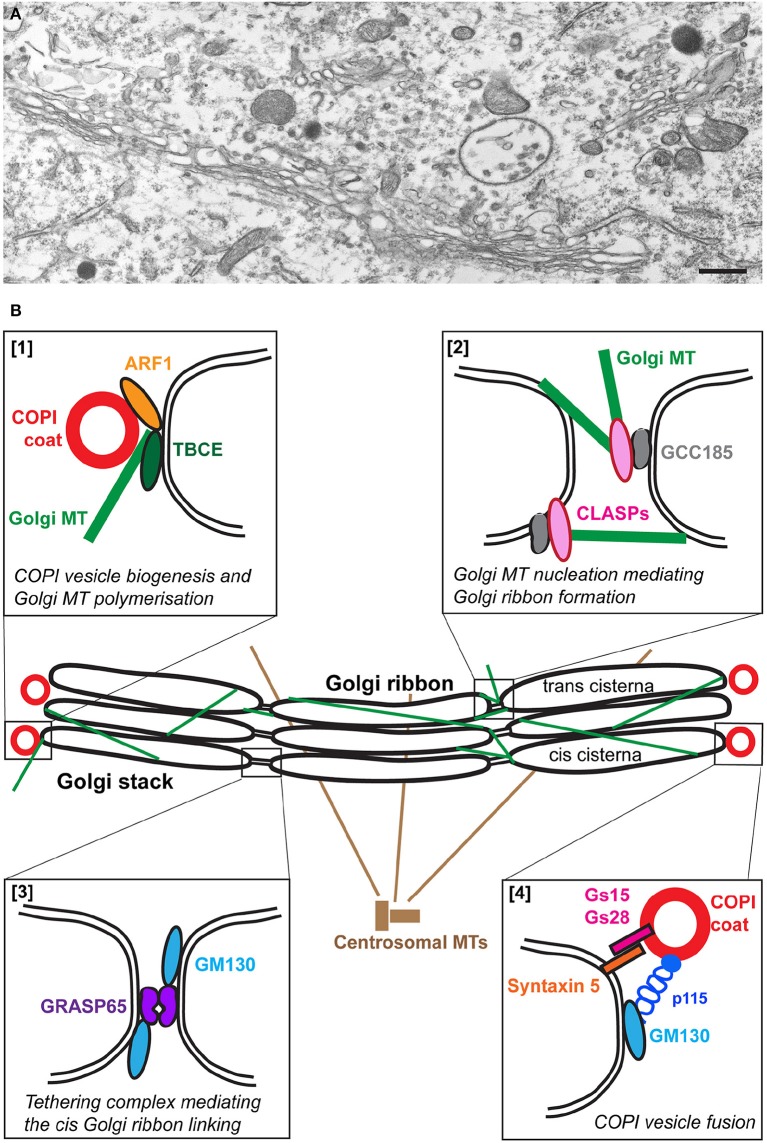
**The Golgi apparatus in wild type motor neurons**. **(A)** An electron micrograph showing part of the typical Golgi ribbon in a mouse lumbar spinal cord motor neuron. **(B)** Schematic representation of some of the molecular players involved in the organization of the wild type Golgi. Polymerization of microtubules (MT) at the cis-Golgi depends on TBCE which mediates cross talk with ARF1-mediated COPI vesicle biogenesis [box 1]. Golgi microtubules nucleated by GCC185/CLASPs at the trans-Golgi play a role in Golgi ribbon linking [box 2]. The formation of the Golgi ribbon at the cis side is mediated by the tethering complex GRASP65/GM130 [box 3]. COPI vesicle fusion is mediated by the tethering complex p115/GM130 and SNARE complexes containing GS15, GS28, and Syntaxin 5 [box4].

The Golgi apparatus is a unique organelle comprising stacks of flattened discrete membrane-bound compartments called cisternae forming the so-called Golgi stacks. In mammalian cells, these stacks are laterally connected by tubules to form a large Golgi ribbon capping the nucleus (Figure 1A) and (Képès et al., 2005; Glick and Nakano, 2009). Furthermore, the Golgi apparatus is polarized with a cis-entry site facing the ER and the ERGIC (ER-Golgi intermediate compartment) and a trans-exit face facing the endosomal system (Polishchuk and Mironov, 2004). In motor neurons, the Golgi apparatus forms a very large network that extends into axons and dendrites (Bellouze et al., 2014; Valenzuela and Perez, 2015).

In motor neurons of ALS patients, the Golgi apparatus often appears either fragmented, i.e., transformed into multiple disconnected elements or tubular-vesicular clusters, or atrophied, i.e., reduced in its membrane content (Mourelatos et al., 1990; Gonatas et al., 1992). These pathological changes are detectable in all types of motor neurons located in spinal cord, brainstem and cerebral cortex (Mourelatos et al., 1996; Fujita et al., 1999, 2000). Furthermore, they are common to both sporadic (Gonatas et al., 1992) and familial (Mourelatos et al., 1996; Fujita et al., 1999, 2000) forms of the disease, including ALS with Bunina bodies (Stieber et al., 1998), juvenile ALS (Fujita et al., 2002), and ALS with posterior column involvement (Fujita et al., 2000).

Golgi fragmentation has been found to be closely associated with other neuropathological hallmarks of ALS, such as cytoplasmic basophilic inclusions (Fujita et al., 2002), ubiquitin aggregates (Vlug et al., 2005), SOD1 aggregates (Fujita et al., 2000) and TDP-43 pathology (Fujita et al., 2008). Importantly, Golgi alterations occur at an early preclinical stage in human ALS patients (Maruyama et al., 2010) and in rodent ALS models (Mourelatos et al., 1996; Vlug et al., 2005; Tong et al., 2012; van Dis et al., 2014), suggesting that Golgi fragmentation precedes the degenerative loss of motor neuron cell bodies and axons. By contrast, conspicuous Golgi alterations in motor neurons are not observed after chemical poisoning (Mourelatos et al., 1994) or axonal injury (Bellouze et al., 2014), ruling out that they are a mere consequence of neuronal damage.

The main function of the Golgi apparatus in healthy cells is to ensure the processing (enzymatic modification and proteolytic cleavage) and sorting of proteins from their site of synthesis in the endoplasmic reticulum to their final destination, plasma membrane, extracellular space and endosomal/lysosomal compartments (Bonifacino and Glick, 2004). The Golgi apparatus also ensures the polarity of protein and lipid transport to axons and dendrites (Tang, 2001; Ramírez and Couve, 2011; Bonifacino, 2014). In particular, the Golgi apparatus is required for the assembly and axonal transport of synaptic vesicle precursors (Maas et al., 2012). Alterations of the Golgi apparatus could therefore be accompanied by loss or gain of function in protein sorting, processing and transport to the axons and synapses, leading to their degeneration. Furthermore, Golgi fragmentation leads to activation of apoptotic pathways (Hicks and Machamer, 2005; Machamer, 2015) that can contribute to axonal degeneration and ultimately to cell death.

Despite their early occurrence and high frequency, the precise relevance of these pathological Golgi alterations remains unclear. Future studies have to clarify whether they have a causative, contributory or homeostatic role in motor neuron degeneration, in particular with respect to the loss of cell bodies, axons and synapses. To be able to address this in a meaningful manner, it is crucial to better understand the precise mechanisms of Golgi pathology in ALS.

## Three classes of proteins are important for golgi maintenance

In theory, Golgi morphology depends on three classes of proteins: The microtubules (MTs) and MT-associated motor proteins; the structural Golgi proteins; and the proteins of the Golgi transport machinery. Of note, most of the knowledge on these alterations comes from studies in mitotic non-neuronal cells and only few data are available from motor neurons.

### The microtubule cytoskeleton

It has been known for 30 years that microtubule depolymerization (for instance with nocodazole or colchicine) alters the Golgi morphology of mammalian cells. Instead of the single copy organelle capping the nucleus in non-treated cells, the Golgi ribbon appears fragmented leading to single stacks or group of stacks (Ellinger and Pavelka, 1984; Rogalski and Singer, 1984; Turner and Tartakoff, 1989; Cole et al., 1996; Scales et al., 1997).

It is also increasingly recognized that in addition to the centrosome, the Golgi is a major site of microtubule formation in various cell types (Chabin-Brion et al., 2001; Efimov et al., 2007; Miller et al., 2009), including hippocampal neurons (Stiess et al., 2010; Yau et al., 2014) and motor neurons (Bellouze et al., 2014), see for review (Zhu and Kaverina, 2013; Rios, 2014; Kapitein and Hoogenraad, 2015; Sanders and Kaverina, 2015).

During neuronal differentiation, the centrosome is dismantled and becomes dispensable for the formation of microtubules and for the establishment of proper axonal and neuronal morphology (Basto et al., 2006; Stiess et al., 2010; Nguyen et al., 2011). The Golgi however expands and rearranges (Horton et al., 2005) and may thus become increasingly for the correct formation, nucleation and dynamics of microtubules.

The formation of microtubules involves the folding of alpha- and beta-tubulins, their dimerization and polymerization. The last steps of this complex process are assisted by five tubulin-specific chaperones termed tubulin-binding cofactors TBCA-TBCE (Tian et al., 1996, 1997). Among these, TBCE is expressed at high levels in motor neurons (Schaefer et al., 2007) where it is associated to the cis-Golgi (Figure 1B) and is critical for the polymerization of Golgi-derived microtubules (Bellouze et al., 2014). The nucleation of Golgi-microtubules then involves the Golgi proteins GM130 (Kodani and Sütterlin, 2008), AKAP-450 (Rivero et al., 2009), and CLASP1/2 (Efimov et al., 2007) (Figure 1B).

### The golgi structural proteins

The second class of proteins that could be affected are the so-called Golgi structural proteins. These are the Golgins, extended coiled-coil proteins that form a proteinaceous matrix around the Golgi (Munro, 2011; Gillingham et al., 2014). Their knock down leads to a structural alteration of the Golgi. This is the case for p115, GM130, but also Golgin 84 (Diao et al., 2003) and Giantin (Koreishi et al., 2013). In addition, two non-Golgin proteins have been shown to be critical for the Golgi architecture, GRASP65 and 55 with a role in the formation of tubular connections between Golgi stacks to form the Golgi ribbon (Vinke et al., 2011; Jarvela and Linstedt, 2012, 2014; Veenendaal et al., 2014; Figure 1B) as well as in cisternal stacking (Xiang and Wang, 2010). Of note, it is sometimes difficult to tease apart the strict role of these proteins in Golgi structure from their role in trafficking thus precisely pin-pointing the primary cause for Golgi fragmentation.

Interestingly, some of these proteins are the targets of specific modifications that occur at the onset of mitosis when the Golgi becomes physiologically fragmented in a controlled and reversible manner. For instance, GM130 is phosphorylated, leading to dissociation of the GM130/p115 complex. As a result, COPI vesicles are no longer tethered and cannot fuse, leading to Golgi fragmentation and generation of mitotic clusters (Nakamura et al., 1997). Furthermore, GM130 (Walker et al., 2004), p115 (Chiu et al., 2002), and GRASP65 (Lane et al., 2002; Cheng et al., 2010) are targets of apoptotic caspase-mediated proteolysis leading to Golgi fragmentation.

### The golgi transport machinery

The third class of proteins that, when altered, could lead to a loss of the typical Golgi architecture are the proteins functioning in the traffic between the ER and the Golgi, especially COPI and COPII coat complexes as well as their tethering/docking/fusion machinery. Trafficking between the ER and the Golgi is mediated by at least two coat complexes.

The COPII coat assembles at specific sites of the ER, called ER exit sites, where COPII coated vesicles form and bud (Miller and Schekman, 2013; Sprangers and Rabouille, 2015). The COPI complex or coatomer assembles at the surface of the Golgi and the ERGIC. The coatomer comprises 6 structural subunits alpha, beta, beta', delta, gamma, and epsilon COP (Beck et al., 2009) and their formation requires the small GTPase Arf1 and its GEF GBF1 (Popoff et al., 2011). There is a strong consensus that COPI coated vesicles mediate the retrograde transport from the Golgi to the ER, but it is also possible that they may also participate in specific anterograde transport steps from the ERGIC to the cis Golgi cisterna and/or within the Golgi cisternae (Aguilera-Gomez and Rabouille, 2015).

After budding from their respective compartments, the COPI and COPII coated vesicles fuse with their target compartments and this requires tethering (p115/GM130), docking (using Rab proteins, Pfeffer, 2013a,b) and fusion proteins, such as the SNAREs. The SNAREs are type II c-tail anchored transmembrane proteins with almost all their mass in the cytoplasm. They belong to two classes, v-SNAREs (present on vesicles) and t-SNAREs (present on target compartments), roughly corresponding to R- and Q-SNAREs. Usually, three Q-SNAREs and one R-SNARE form a tight parallel helix that mediates vesicle fusion (Malsam and Söllner, 2011).

Impairment of the balance between vesicle budding and fusion either at the Golgi or the ER leads to a loss of Golgi architecture and its complete vesiculation. For instance, when COPII vesicle budding is inhibited, the Golgi vesiculates (Prescott et al., 2001). The Golgi appears similarly vesiculated when COPI formation is impaired by BFA treatment or depletion of COPI subunits (Guo et al., 2008; Razi et al., 2009). Last, impaired Golgi SNARE function like depletion of Syntaxin 5 (Suga et al., 2005) also leads to strong Golgi fragmentation, as observed in cellular models of Parkinson disease (Rendón et al., 2013).

Furthermore, at least one caspase (caspase 2) is localized to the Golgi (O'Reilly et al., 2002), suggesting that the Golgi can sense cell death signals and transduce them via cleavage of structural Golgi proteins (Galluzzi et al., 2014).

## Proposed triggers of golgi fragmentation in ALS and related motor neuron diseases

The precise mechanisms of Golgi fragmentation in degenerating motor neurons are not fully understood but first insights arise from studies in animal and cellular models of ALS and related motor neuron diseases linked to mutations in SOD1, Dynein/Dynactin, TARDBP (TDP-43), and VAP-B.

Schematically, four mechanisms of Golgi fragmentation can be distinguished: The first one involves defective microtubules or microtubule-dependent transporters due to mutations in SOD1 (Soo et al., 2015), Dynein/Dynactin (Hafezparast et al., 2003; Puls et al., 2003), and BICD2 (Neveling et al., 2013; Peeters et al., 2013). Dynein and BICD2 will not be discussed further as two excellent reviews have just been published on that topic (Jaarsma and Hoogenraad, 2015; Wirth and Martinez-Carrera, 2015). The second one postulates the cleavage of Golgi structural proteins by activation of caspases (SOD1). The third and fourth ones hypothesize that Golgi fragmentation is due to toxic protein aggregates (TDP-43, SOD1) or dysfunctional proteins (VAP-B), which potentially impair trafficking within the early secretory pathway.

Here, we first summarize these data before describing a novel mechanism that involves both microtubules, Golgi vesicles and structural Golgi proteins, and which is potentially shared by several forms of human ALS, spinal muscular atrophy (SMA), and related motor neuron diseases.

### Microtubule disruption by mutant SOD1

Mutations in the human SOD1 (Cu/Zn superoxide dismutase 1) gene account for ~5% of familial forms of ALS (Andersen and Al-Chalabi, 2011) and are at the origin of the most widely used animal and cellular models of ALS. Transgenic mice expressing ALS-linked SOD1 mutations manifest signs of motor neuron degeneration such as early axonal dying back and loss of neuromuscular synapses (Fischer et al., 2004; Schaefer et al., 2005; Pun et al., 2006). In these mice, Golgi fragmentation occurs at an early preclinical stage and precedes most other histopathological alterations (Mourelatos et al., 1996; Vlug et al., 2005; van Dis et al., 2014).

Earlier studies suggested that mutant SOD1-linked Golgi fragmentation may originate from microtubule alterations. Indeed, the mean diameter of Golgi cisternae was found to be shorter in mice overexpressing mutant SOD1 G93A when compared to those expressing wildtype SOD1 (Gonatas et al., 1992), an effect similar to that of the microtubule-disrupting drug colchicine (Mourelatos et al., 1990). A recent study further shows that cellular overexpression of mutant SOD1 is associated with unstable microtubules and decreased levels of acetylated tubulins (Soo et al., 2015).

Furthermore, the microtubule-severing protein Stathmin-1 of the Stathmin family that regulates microtubule transition from growing to shrinking phases, some of which are also associated with the Golgi membrane (Gavet et al., 1998) has been found to be up-regulated both at the RNA (Ferraiuolo et al., 2007) and the protein (Strey et al., 2004) level in mutant SOD1-expressing mouse motor neurons. Finally, the most acidic isoform of Stathmin-1, which probably corresponds to the inactive phosphorylated protein, was found selectively down-regulated (Strey et al., 2004).

However appealing, this hypothesis is not yet proven since no conspicuous microtubule alterations have been detected in mutant SOD1 motor neurons with a fragmented Golgi following standard tissue preparation (Strey et al., 2004). To detect SOD1-triggered microtubule alterations *in vivo*, better techniques for microtubule preservation and visualization such as high pressure freezing and EM-based 3D reconstruction (Marsh et al., 2001) may be required. To firmly establish the role of Stathmin-1 and the related Stathmins 2–4 as potential mediators of mutant SOD1-triggered Golgi pathology, it will be necessary to recapitulate Golgi fragmentation by Stathmin overexpression and to rescue mutant SOD1-linked Golgi fragmentation by Stathmin knock down.

### Caspase-mediated cleavage of structural golgi proteins by mutant SOD1

Mutant SOD1-linked Golgi fragmentation in motor neurons may involve defects in structural Golgi proteins that have been shown to be targets of caspases as described above. We have previously shown that mutant SOD1 sensitizes motor neurons to Fas-triggered cell death involving caspases 8 and 3 (Raoul et al., 2002, 2006). Additional studies have shown that caspases 1, 3, 9, and 12 are activated in mutant SOD1 spinal cord (Li et al., 2000; Inoue et al., 2003; Kikuchi et al., 2006). Whether mutant SOD1 triggers caspase-cleavage of Golgi proteins and thereby contributes to Golgi fragmentation in motor neurons remains however to be investigated.

### Protein aggregates and golgi fragmentation: TDP-43

Typical ALS is characterized by cytoplasmic inclusions made of irreversible protein aggregates comprising the nuclear transactivation response (TAR) DNA-binding protein of 43-kDa (TDP-43; Neumann et al., 2006). Furthermore, TARDBP (TDP-43) mutations are associated with sporadic and familial forms of ALS (Sreedharan et al., 2008; Van Deerlin et al., 2008) and were reported to form cytosolic protein aggregates in human postmortem tissues (Lee et al., 2012) and in iPSc-derived motor neurons (Egawa et al., 2012) of patients. The TDP-43 protein aggregates occur in many, albeit not all, neuronal types in patients (Toyoshima and Takahashi, 2014) and in animal models (Tsao et al., 2012). The formation of these inclusions is thought to impair TDP-43 critical nuclear function in neuronal RNA metabolism, including the splicing and stability of numerous RNAs encoding proteins involved in neuronal development, synaptic function and neurodegeneration (Lagier-Tourenne et al., 2012; Jovicic and Gitler, 2014; Lee et al., 2015; Ludolph and Brettschneider, 2015; Scotter et al., 2015; Smethurst et al., 2015). Thus, a loss of these functions through cytoplasmic irreversible protein aggregation is an attractive hypothesis regarding the role of TDP-43 in neurodegeneration (Dewey et al., 2012).

Interestingly, the presence of TDP-43 positive cytoplasmic inclusions has been associated with Golgi fragmentation in spinal motor neurons of ALS patients (Fujita et al., 2008). Similarly, in transgenic rats overexpressing mutant human TDP-43, Golgi fragmentation is detected in a concomitant manner with the formation of TDP-43 aggregates (also positive for ubiquitin) in the same neuron (Tong et al., 2012). Whether and how cytoplasmic TDP-43 protein aggregates are mechanistically linked to Golgi fragmentation is however not known, and the potential Golgi targets and stress signaling pathways of TDP-43 inclusions remain to be identified.

### Golgi fragmentation due to impairment in the early secretory pathway trafficking

#### Aggregated SOD1 and golgi fragmentation

Overexpressed mutant SOD1 in CHO cells is also shown to aggregate, thus trapping key proteins of the early secretory pathway (see below Atkin et al., 2014). In contrast, mutant SOD1 is seen diffuse in the cytoplasm in Neuro2a cells but its aggregation is triggered upon overexpression of chromogranins, components of neurosecretory vesicles. Interestingly, this aggregation pattern is consistent with the Golgi/TGN that appears disrupted in large vesicles (Urushitani et al., 2006), a result that can be explained by the interaction between chromogranins and mutant SOD1.

This suggests that mutant SOD1 is recruited to the Golgi through its interactions with chromogranins. This could be consistent with an association of SOD1 on the cytoplasmic leaflet of the Golgi membrane, but the authors show that mutant SOD1 is secreted to the extracellular medium together with the chromogranins. This suggests that mutant SOD1 has somehow reached the lumen of these large vesicles, an event proposed to occur early in the early secretory pathway (Urushitani et al., 2008). In this specific case, however, the observed Golgi disruption is not due to expression of mutant SOD1, but to the co-expression of chromogranins. The mechanism for the chromogranin-mediated mutant SOD1 translocation remains to be elucidated.

Several studies also indicate that misfolded mutant forms of SOD1 can impede ER-to-Golgi transport (Stieber et al., 2004). Indeed, using NSC-34 motor neurons cell in culture, expression of mutant SOD1 was shown to first inhibit ER-Golgi trafficking, an event that preceded ER stress (Turner and Atkin, 2006; Atkin et al., 2014), and Golgi fragmentation. Interestingly, four different mutant forms of SOD1 (but not wildtype SOD1) have been shown to bind the COPII subunit Sec23 that co-localizes with aggregated mutant SOD1. This presumably renders it non-functional (Atkin et al., 2014) and leads to Golgi fragmentation that was rescued upon overexpression of the small GTPase Sar1. The interaction between mutant SOD1 and Sec23 was also reported in SOD1 mouse spinal cord at the early age of 10 days. These findings may thus link Golgi fragmentation to the early disruption of ER exit in mutant SOD1 expressing cells (Atkin et al., 2014).

#### Loss VAP-B induces golgi fragmentation

Atypical forms of familial ALS (ALS8) and late-onset SMA have been associated with a dominant missense mutation (P56S) in the VAPB gene (Nishimura et al., 2004, 2005; Marques et al., 2006; Funke et al., 2010). Additional VAP-B mutations have reported been in few patients with typical ALS (Kabashi et al., 2013).

VAP-B and its homolog VAP-A are members of the highly conserved and ubiquitously expressed VAP (Vesicle-Associated Membrane Protein (VAMP)-Associated Protein) family of ER C-tail anchored proteins. This class of proteins has been shown to interact with the FFAT motif (two phenylalanines in an acidic tract) characteristic of lipid transfer proteins enriched at membrane contact sites between the ER and the Golgi (Mesmin et al., 2013) and other organelles (Levine and Loewen, 2006). In addition, VAP proteins seem implicated in membrane trafficking, ER/cytoskeleton interactions, the unfolded protein response (reviewed in (Lev et al., 2008), calcium homeostasis (De Vos et al., 2012; Stoica et al., 2014), axonal transport of mitochondria (Mórotz et al., 2012) as well as neurite extension and neurotransmitter release (Saita et al., 2009; Ohnishi et al., 2014).

When overexpressed, the ALS-linked VAP-B mutant P56S favors the formation of clusters of paired ER cisternae (Fasana et al., 2010; Kuijpers et al., 2013b) that are located close to the Golgi (Genevini et al., 2014). This leads to ER stress and disrupts ER to ERGIC to Golgi trafficking (Kuijpers et al., 2013b). It also causes Golgi fragmentation in a small number (15%) of primary rat hippocampal neurons (Teuling et al., 2007), although this was not observed (albeit not quantified) in spinal motor neurons on sections (Kuijpers et al., 2013a) or in cell culture (Genevini et al., 2014).

One reason is that overexpression of P56S VAP-B does not completely recapitulate the ALS situation. In fact, P56S VAP-B leads to the degradation of wildtype VAP-B (and probably VAP-A) and a consequent reduced level of the functional protein (Suzuki et al., 2009; Papiani et al., 2012) but not the formation of aberrant ER structures. Similarly, the endogenous mutant P56S allele causes a reduction of VAP-B levels in patient-derived iPSc motor neurons (Mitne-Neto et al., 2011) and its fly equivalent P58S leads to aggregation of wildtype VAP-B (Ratnaparkhi et al., 2008). This is also observed in Vapb P56S knockin mice (Larroquette et al., 2015) where cleaved ubiquitinated VAP-B accumulates in insoluble complexes.

Loss of VAP-B function may also be involved in other forms of ALS since reduced VAP-B levels have been reported in the spinal cord of mutant SOD1 mice and of patients with sporadic ALS (Teuling et al., 2007; Anagnostou et al., 2010). Furthermore, overexpression of VAP-B P56S in mice leads to pathological TDP-43 aggregates both in the nucleus and the cytoplasm of motor neurons (Tudor et al., 2010), which, as reported above, may contribute to disruption of ER to Golgi transport.

Importantly, VAP-B depletion in HeLa cells leads to strong Golgi fragmentation (Peretti et al., 2008). This is likely due to the pleiotropic effects of VAP-B on Golgi-mediated transport pathways (Peretti et al., 2008) where it interacts with numerous Golgi-associated proteins (Huttlin et al., 2015) and microtubules (Skehel et al., 2000). ER stress and autophagy (Larroquette et al., 2015) may be further involved in pathogenesis.

## A novel mechanism of golgi fragmentation: defective cross talk between the microtubule network and COPI vesicles

### Progressive motor neuronopathy (pmn)

Recently, studies in *pmn* mice carrying a missense mutation in the tubulin-binding cofactor TBCE have led to the elucidation of a mechanism where microtubules, structural Golgi proteins and the Golgi transport machinery are altered, and illustrated a molecular cross talk between these three classes of proteins (Bellouze et al., 2014). Loss of TBCE function in *pmn* mice causes axonal dying back of motor neurons (Schaefer et al., 2007) and loss of cochlear outer hair cells (Rak et al., 2013), growth and mental retardation in human patients with Sanjad-Sakati/Kenny-Caffey syndrome (Parvari et al., 2002) and impaired development of motor axons and neuromucular synapses in Drosophila (Jin et al., 2009). Both the TBCE *pmn* mutation, which leads to protein destabilization and degradation (Martin et al., 2002; Schaefer et al., 2007), and cellular TBCE depletion (Bellouze et al., 2014) result in a general loss of microtubules including Golgi-derived ones.

**Figure 2:**
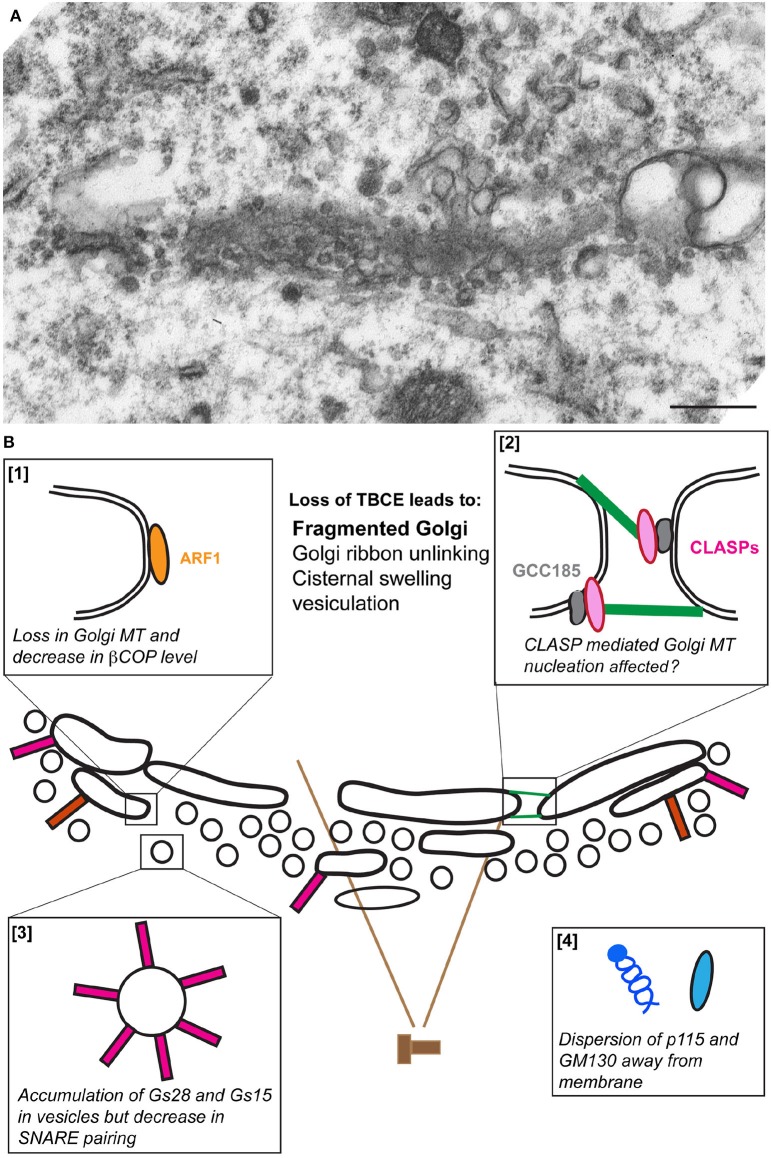
**The fragmentation of the Golgi apparatus in *pmn* motor neurons**. **(A)** An electron micrograph showing vesiculation of the Golgi stack in a lumbar spinal cord motor neuron from a *pmn* mouse aged 35 days. **(B)** Schematic representation of the molecular defects leading to Golgi fragmentation. Loss of TBCE function impedes polymerization of microtubules at the cis-Golgi [box 1] and potentially also their GCC185/CLASP-dependent nucleation at the trans-Golgi [box 2]. Levels of β-COP and ε-COP are reduced [box 1], vesicles containing high amounts of Golgi SNAREs GS15 and GS28 accumulate [box3] and tethers p115 and GM130 disperse away from membranes [box 4].

Mutant *pmn* mice display clear signs of Golgi fragmentation in lumbar spinal motor neurons at 10 days of age i.e., 5 days before first clinical, electrical or pathological signs of axon degeneration (Schmalbruch et al., 1991; Kennel et al., 1996; Bellouze et al., 2014), confirming the early preclinical occurrence of structural Golgi alterations. During disease course, Golgi membranes are progressively transformed into vesicles and tubules (Figure 2A) and the percentage of motor neurons with signs of Golgi fragmentation increases massively (Bellouze et al., 2014).

Biochemical and microscopic analysis of *pmn* associated Golgi defects shows three critical aspects of Golgi fragmentation: a strong decrease in the protein levels of the beta and epsilon COPI vesicle subunits, a dispersion of the Golgi tethers p115 and GM130 away from Golgi membrane and an about 15-fold increase in the protein levels of Golgi v-SNAREs GS15 and GS28 (Bellouze et al., 2014). This accumulation is due to protein stabilization rather than transcriptional up-regulation, and is nevertheless associated with compromised formation of v-/t-SNARE complexes (Bellouze et al., 2014; Figure 2B).

Taken together, loss of TBCE leads to loss of Golgi-associated microtubules, a fragmented Golgi and associated molecular defects reported above. This suggests that defects in TBCE-mediated microtubule polymerization at the Golgi impede the COPI coat assembly that in turn causes mislocalization of associated tethers and accumulation of Golgi SNAREs resulting in Golgi fragmentation. This scenario of Golgi fragmentation is further supported by reports showing that depletion of β-COP causes accumulation of small vesicles containing Golgi enzymes at the cell center (Guo et al., 2008) and redistribution of GM130 (Razi et al., 2009), potentially through dysfunction of the ER/Golgi intermediate compartment (ERGIC; Horstmann et al., 2002; Xu and Hay, 2004).

Intriguingly, COPI dynamics feeds back on microtubule polymerization at the Golgi. Expression of constitutionally active GTP-locked ARF1 following TBCE knock down (not knock out) stimulates recruitment of remaining TBCE to the Golgi, polymerization of microtubules and rescue of the Golgi defects (Bellouze et al., 2014). This may be due to an interaction between Arf1 and TBCE, at least upon overexpression of both proteins, making TBCE an Arf1 effector. We therefore propose that the cross talk between microtubules in the cell soma and COPI vesicle dynamics at the Golgi maintains the Golgi structure and that the interruption of this cross talk causes microtubule disruption and Golgi fragmentation observed in *pmn* mice.

### Other mouse mutants with motor neuron degeneration

Is the interruption of the cross talk also the mechanism of Golgi fragmentation in other disease models? Microtubule and COPI defects may indeed contribute to Golgi fragmentation in *mdf (muscle deficient)* mice mutated in the Scyl1 gene. Scyl1 encodes a protein that normally interacts with α-COP and β-COP (Burman et al., 2008, 2010), suggesting that Golgi fragmentation in motor neurons of *mdf* mice involves defective COPI vesicle formation which may interrupt the COPI/MT cross talk with ensuing Golgi fragmentation.

Furthermore, SNARE-mediated vesicle tethering may be defective in *wobbler* mice with motor neuron degeneration. Indeed the mutant gene product VPS54 (Schmitt-John et al., 2005) is a subunit of the Golgi-associated retrograde protein (GARP) complex that normally tethers vesicle budding from late endosomes to the TGN through interaction with the v-SNAREs Syntaxin-6, Syntaxin-16, and VAMP4 (Pérez-Victoria et al., 2008; Pérez-Victoria and Bonifacino, 2009).

### Human ALS linked to TUBA4A mutations

Rare forms of human ALS have recently been associated with mutations in the tubulin gene TUBA4A that encodes a major alpha-tubulin isoform of adult spinal cord and brain (Smith et al., 2014). *In vitro* overexpression of several ALS-linked TUBA4A mutations causes severe alterations in the somatic microtubule network (Smith et al., 2014). Additional forms of human ALS and SMA are due to mutations in the microtubule motors Dynein (Hafezparast et al., 2003), its regulator BICD2 (Neveling et al., 2013; Peeters et al., 2013), or in Optineurin (Maruyama et al., 2010), a protein known to bind microtubules and to mediate vesicle transport in the secretory pathway. So, defective microtubule-dependent transport as well as impairment in trafficking through the Golgi may be the basis for Golgi fragmentation in these diseases.

### SMA

Defective cross talk between microtubules and COPI vesicles may also be involved in classical SMA caused by deletion of the SMN (Survival Motor Neuron) gene (Lefebvre et al., 1995). Although Golgi fragmentation has not yet been reported in SMN-linked SMA, increased levels of Stathmin-1 (see above) are associated with microtubule loss, axon degeneration and loss of neuromuscular synapses in mouse and cellular models of SMA (Wen et al., 2010, 2013). Furthermore, SMN has been shown to bind the COPI subunit α-COP and to co-traffic with the latter in motor axons (Peter et al., 2011). Interrupting SMN/α-COP interaction leads to SMN accumulation at the Golgi (Ting et al., 2012), reduced neurite outgrowth and altered growth cones (Custer et al., 2013). We therefore hypothesize that the Golgi is fragmented in SMA mice and patients through SMN/COPI/microtubule dysfunction.

### ALS linked to C9Orf72 mutations

The most frequent forms of human ALS are caused by novel GGGGCC (G_4_C_2_) hexanucleotide repeat expansions in the first intron of the C9Orf72 gene (DeJesus-Hernandez et al., 2011; Renton et al., 2011). Patients with this form of ALS typically carry several hundred to more than thousand G_4_C_2_ repeats (Beck et al., 2013; Dols-Icardo et al., 2014), although symptoms may be caused by as few as 20 G_4_C_2_ repeats (Gómez-Tortosa et al., 2013).

According to the main current hypotheses, G_4_C_2_ repeat expansions may cause disease through formation of G-quadruplex RNA structures leading to nucleolar stress (Haeusler et al., 2014), formation of nuclear RNA foci sequestering essential RNA-binding proteins (DeJesus-Hernandez et al., 2011; Polymenidou et al., 2012), repeat-associated non-AUG (RAN) unconventional translation into toxic poly-dipeptides (DPRs; Cleary and Ranum, 2013; Mori et al., 2013; Zu et al., 2013), or by impeding nucleocytoplasmic transport (Freibaum et al., 2015; Jovicic et al., 2015; Zhang et al., 2015).

It should be stressed that Golgi fragmentation has not yet been reported in mutant C9Orf72-linked ALS. However, several findings point to a role of the wildtype C9ORF72 protein in vesicle trafficking. Indeed, bioinformatic analyses predict the presence of DENN (differentially expressed in normal and neoplastic cells) domains in C9Orf72 (Zhang et al., 2012; Levine et al., 2013). DENN domains bear sequence and structural homology with guanine nucleotide exchange factors (GEFs) for Rab GTPases, some of which are involved in regulating vesicle trafficking at the Golgi (Liu and Storrie, 2012). A recent study has reported that the C9Orf72 protein asssociates with Rab1, Rab7 and Rab11 (Farg et al., 2014). In addition to impairment of autophagy and endocytic trafficking upon C9ORF72 knockdown, the GM130 pattern is altered potentially consistent with Golgi alterations (Farg et al., 2014).

Since gain of function rather than loss of function mechanisms appear to drive motor neuron degeneration in C9Orf72-linked ALS (Koppers et al., 2015), future studies need to address the toxic consequences of G_4_C_2_ repeat expansions and DPRs on vesicle trafficking and maintenance of the Golgi apparatus. The G_4_C_2_ repeat expansions can be associated with the formation of TDP-43 nuclear and cytoplasmic inclusions (Mori et al., 2013; but see Al-Sarraj et al., 2011), which possibly affects ER to Golgi transport. In addition, the C9Orf72 ALS-associated DPR Gly-Ala has recently been shown to sequester the cargo adaptor Unc119/HRG4 into protein aggregates (May et al., 2014). This may disrupt the ternary complex that is normally formed by Unc119/HRG4, the tubulin-binding co-factor D (TBCD) and the ARF-subfamily member ARL2 (Veltel et al., 2008; Ismail et al., 2012). These data suggest that defects in the MT/COPI cross talk may be implicated in this frequent form of human ALS.

## What is the relevance of golgi fragmentation to motor neuron degeneration?

While Golgi fragmentation is now recognized as an early and constant hallmark of degenerating motor neurons in ALS and related motor neuron diseases, its relationship to the neurodegenerative process remains to be clarified. Numerous studies indicate that structural and functional Golgi alterations are not simply a consequence of neurodegeneration. For instance, the drug β,β′-iminodiproprionitrile (IDPN) that causes severe proximal axonopathy does not induce any morphometric changes of the Golgi in rat motor neurons (Mourelatos et al., 1994). Furthermore, surgical transection of the rat facial nerve does not trigger the fine Golgi fragmentation typically observed in human ALS patients (Fujita et al., 2011). Last, transection of the sciatic nerve in mice does not alter the cellular levels or distribution of β-COP, the Golgi tether p115 or the Golgi SNAREs GS28 and GS15 in the corresponding motor neuron cell bodies (Bellouze et al., 2014), suggesting that the microtubule/COPI cross talk is unaffected by axonal injury, in contrast to the situation in *pmn* mice.

Conversely, mounting evidence suggests that Golgi alterations may contribute to, or in some cases even cause, aspects of motor neuron degeneration. In particular, alterations in the microtubule/COPI cross talk may impact on the normal function of the Golgi apparatus in the transport of proteins, lipids and RNAs that are essential for axon and synapse maintenance. The COPI subunits α-, β-, and γ-COP for instance bind numerous mRNAs (Bi et al., 2007; Todd et al., 2013) and facilitate their axonal transport along microtubules (Bi et al., 2007). Furthermore, ARF1 function is critical for axon growth and maintenance (Jareb and Banker, 1997), suggesting a role of COPI in vesicle biogenesis and/or axonal transport.

In conclusion, more work is needed to understand the relevance of Golgi fragmentation for onset and progression of motor neuron degeneration. One way forward is to experimentally inhibit Golgi fragmentation in model systems, whether *in vitro* or *in vivo*, and to subsequently assess the specific consequences on axon degeneration, loss of synaptic vesicles and synaptic dysfunction. To achieve this, we need to gain deeper insights into the mechanisms driving this fragmentation.

Defects in the cross talk between the Golgi microtubule network and the Golgi transport COPI machinery seem an early common mechanism shared by human ALS, SMA, and other motor neuron diseases and thus appear as exciting new molecular targets for the development of biomarkers and pharmacological or gene-based therapies in these severe and currently incurable disorders.

## Author contributions

GH and CR prepared and wrote this manuscript.

### Conflict of interest statement

The authors declare that the research was conducted in the absence of any commercial or financial relationships that could be construed as a potential conflict of interest.
